# PLoS Biology 2.0

**DOI:** 10.1371/journal.pbio.0060048

**Published:** 2008-02-26

**Authors:** Jonathan A Eisen

## Abstract

Jonathan Eisen discusses his commitment to open access publishing, and his plans as the first Academic Editor-in-Chief of PLoS Biology.

In 2003, I was invited to be an Academic Editor for *PLoS Biology* before it published its first article. I was generally supportive of the open-access (OA) movement that PLoS helped foment. But OA publishing in biomedicine was in its infancy, and it was unclear how and if it would succeed. The question that most interested me was whether researchers would choose OA venues for high-profile papers that could have been published in the “big” closed-access journals. I was struggling with this issue myself, as I was working in a hot field (genome sequencing) whose papers were being actively solicited by journals like *Nature* and *Science*. Convincing myself and colleagues to forgo the potentially career-building prestige of these venerable publishing titans to “do the right thing” and go with new OA journals was tough. Thus, *PLoS Biology*, with its goal of being an OA venue for top-tier science, had great appeal.

So I accepted the invitation and became an Academic Editor. But I confess that I was not yet a true convert to OA or to *PLoS Biology*. So I decided to do what any good scientist should do in such a situation—I planned a publishing experiment. I'd had many papers in *Science* and *Nature* before. And so I convinced my collaborators on a high-profile paper to submit it to *PLoS Biology*, to see how this new high-profile OA journal would compare.

But then, while finalizing the paper, a two-month-long medical nightmare ensued that eventually ended in the stillbirth of my first child. While my wife and I struggled with medical mistakes and negligence, we felt the need to take charge and figure out for ourselves what the right medical care should be. And this is when I experienced the horror of closed-access publishing. For unlike my colleagues at major research universities that have subscriptions to all journals, I worked at a 300-person nonprofit research institute with a small library. So there I was—a scientist and a taxpayer—desperate to read the results of work that I helped pay for and work that might give me more knowledge than possessed by our doctors. And yet either I could not get the papers or I had to pay to read them without knowing if they would be helpful. After we lost our son, I vowed to never publish in non-OA journals if I was in control.

When I returned to work, we submitted our paper to *PLoS Biology*, and it was accepted and published in 2004. My OA conversion meant that I was no longer looking at this as an experiment. But it was still an experiment for my collaborators, as well as for colleagues who were skeptical of *PLoS Biology*. And contrary to the dire predictions of some, the experience was spectacular. Not only did we get the same press coverage and scientific acclaim as with other high-profile papers we had published, but we also got attention from people outside of our field, from nonscientists, and even from a neighbor or two.

In the four years since we published that paper, *PLoS Biology* has rapidly proven that OA and “top tier” can go hand-in-hand, thanks to the combined efforts of its staff and Academic Editors and the scientists who have chosen to publish in the journal. And as *PLoS Biology* has thrived, it has become the central icon of PLoS and OA, lending its prestige to PLoS's other ventures, including community-run journals and now *PLoS ONE*.

But that is the past. The key question now is—where does *PLoS Biology* go from here? It is from a desire to help answer that question that I have agreed to serve as Academic Editor-in-Chief. In this role I have three main goals.

First, I want to work to preserve and improve upon the partnership between academic and professional editors that makes *PLoS Biology* different from other top-tier journals (see the accompanying Editorial by the *PLoS Biology* Editors for more details on how *PLoS Biology* works). This partnership presents many difficulties in running the journal and plotting its future, as unlike society journals or other specialty journals, *PLoS Biology* Academic Editors are from all over the map, literally and figuratively. But what can unite the Academic Editors is OA itself, and I believe that OA provides a powerful bridge to get the Academic Editor community more involved in the journal beyond just shepherding papers.

Second, I want to work with the professional staff at *PLoS Biology*, the Academic Editors, and anyone else in the community who shares my desire to build new initiatives that will keep *PLoS Biology* as a top-tier journal. These would include ideas like producing issues dedicated to particular themes, actively recruiting excellent papers in fields where OA is not yet common, producing more outreach and educational material, and engaging bloggers and fully embracing the Web 2.0 world.

Finally, I want to leverage *PLoS Biology*'s position as one of the best and best-known OA journals to energize the OA movement itself. The Creative Commons licenses that PLoS journals use (see http://journals.plos.org/plosbiology/license.php) provide a wealth of benefits to users—who are restricted only by their creativity in how they can use *PLoS Biology*'s contents. I am particularly interested in promoting ways for educators to take full advantage of the benefits of unrestricted, free access to scientific publications. I would like to see *PLoS Biology* contribute to educational initiatives not just by producing material itself but also by demonstrating innovative applications of Creative Commons material in science education—surely a boon to overburdened, under-resourced teachers everywhere. I also believe that *PLoS Biology* can help provide more direct benefits to those who choose to publish in OA journals by lobbying university promotion and hiring committees, funding agencies, and others to encourage OA publishing and to reward it. Given that there are various inducements for other aspects of open science (e.g., many funding agencies require open data release and encourage making software open source), why should there not be rewards for OA publishing?

In the end, I simply love *PLoS Biology* and OA publishing. I am looking forward to my new role at *PLoS Biology*, and I hope to help further its success and that of the OA movement.

**Figure:**
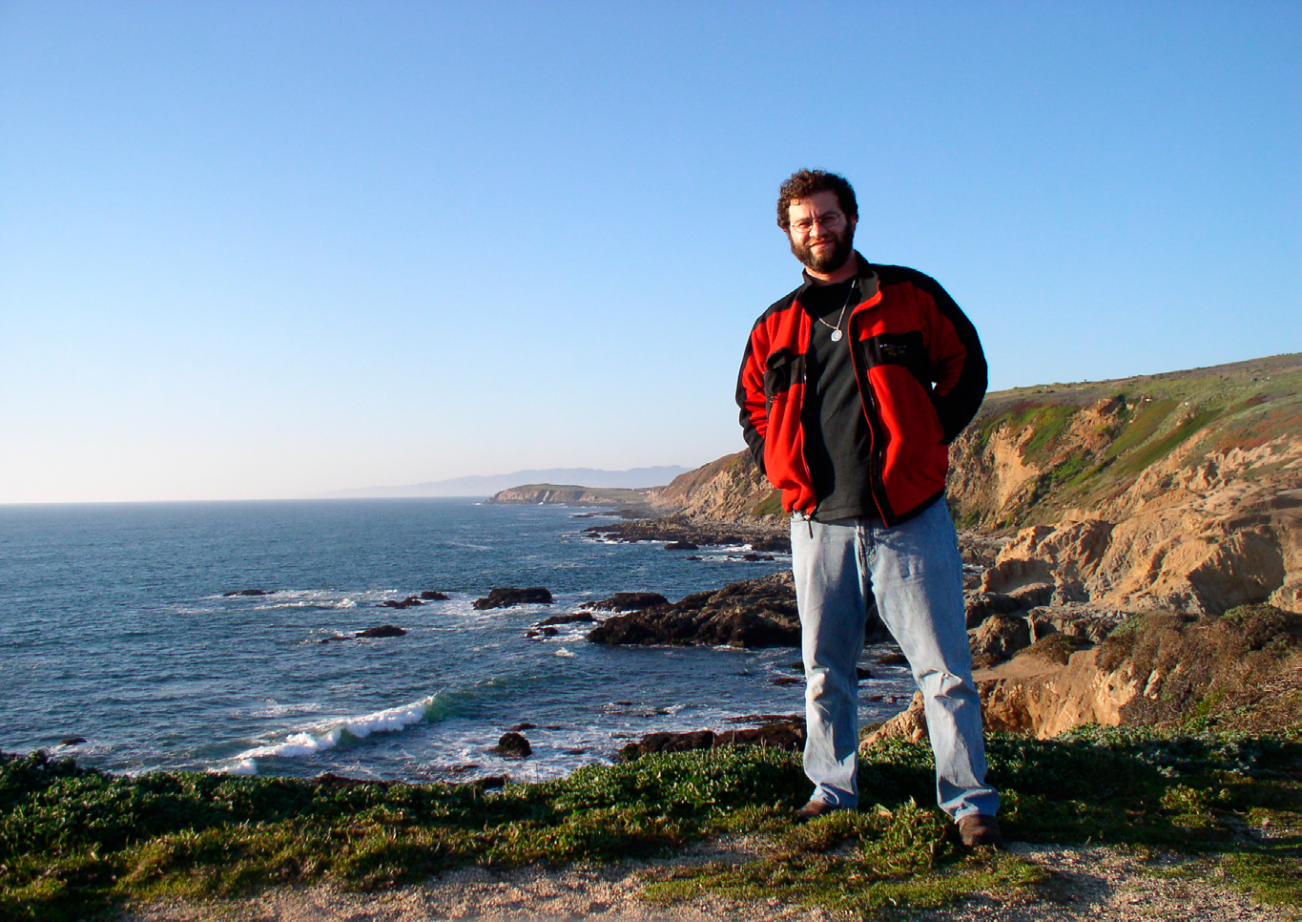
Jonathan Eisen

